# Matrix Metalloproteinase 8 Expression in a Tumour Predicts a Favourable Prognosis in Pancreatic Ductal Adenocarcinoma

**DOI:** 10.3390/ijms23063314

**Published:** 2022-03-18

**Authors:** Mirjami Kaasinen, Jaana Hagström, Harri Mustonen, Timo Sorsa, Malin Sund, Caj Haglund, Hanna Seppänen

**Affiliations:** 1 Department of Surgery, University of Helsinki and Helsinki University Hospital, 00290 Helsinki, Finland; mirjami.kaasinen@helsinki.fi (M.K.); harri.mustonen@helsinki.fi (H.M.); malin.sund@helsinki.fi (M.S.); caj.haglund@helsinki.fi (C.H.); 2 Department of Pathology, University of Helsinki and Helsinki University Hospital, 00290 Helsinki, Finland; jaana.hagstrom@hus.fi; 3 Department of Oral Pathology and Radiology, University of Turku, 20014 Turku, Finland; 4 Translational Cancer Medicine Research Programme, Faculty of Medicine, University of Helsinki, 00290 Helsinki, Finland; 5 Department of Oral and Maxillofacial Diseases, University of Helsinki and Helsinki University Hospital, 00290 Helsinki, Finland; timo.sorsa@helsinki.fi; 6 Section of Periodontology and Dental Prevention, Division of Oral Diseases, Department of Dental Medicine, Karolinska Institutet, 17177 Solna, Sweden; 7 Department of Surgery and Perioperative Sciences/Surgery, Umeå University, 90187 Umeå, Sweden

**Keywords:** pancreatic cancer, matrix metalloproteinase 8, polymorphonuclear cell, C-reactive protein

## Abstract

Pancreatic ductal adenocarcinoma (PDAC) is a significant cause of cancer-related death globally, and, despite improvements in diagnostics and treatment, survival remains poor. Matrix metalloproteinases (MMPs) are enzymes involved in stroma remodelling in inflammation and cancer. MMP-8 plays a varied prognostic role in cancers of the gastrointestinal tract. We examined the prognostic value of MMP-8 immunoexpression in tumour tissue and the amount of MMP-8-positive polymorphonuclear cells (PMNs) in PDAC and their association with immune responses using C-reactive protein (CRP) as a marker of systemic inflammation. Tumour samples from 141 PDAC patients undergoing surgery in 2002–2011 at the Department of Surgery, Helsinki University Hospital were stained immunohistochemically, for which we evaluated MMP-8 expression in cancer cells and the amount of MMP-8-positive PMNs. We assessed survival using the Kaplan–Meier analysis while uni- and multivariable analyses relied on the Cox proportional hazards model. A negative MMP-8 stain and elevated CRP level predicted a poor prognosis (hazard ratio [HR] = 6.95; 95% confidence interval (CI) 2.69–17.93; *p* < 0.001) compared to a positive stain and low CRP level (<10 mg/L). The absence of PMNs together with an elevated CRP level also predicted an unfavourable outcome (HR = 3.17; 95% CI 1.60–6.30; *p* = 0.001). MMP-8 expression in the tumour served as an independent positive prognostic factor (HR = 0.33; 95% CI 0.16–0.68; *p* = 0.003). Tumour MMP-8 expression and a low CRP level may predict a favourable outcome in PDAC with similar results for MMP-8-positive PMNs and low CRP levels. Tumoural MMP-8 expression represents an independent positive prognostic factor in PDAC.

## 1. Introduction

Pancreatic ductal adenocarcinoma (PDAC) is an aggressive disease with a 5-year survival of less of than 10% [1,2]. PDAC stands as the twelfth most common cancer and the seventh most common cause of cancer death globally [3]. Most patients present with metastatic or locally advanced disease, whereby the poor prognosis can be partially explained by the late diagnosis. Surgical resection combined with adjuvant systemic treatment is the only curative treatment, albeit an option for only a minority of patients [4,5]. Earlier detection of PDAC is required to improve patient outcomes.

In addition to challenges in diagnostics, PDAC’s aggressive pathophysiology complicates disease management. Mutations to various genes, tumour heterogeneity and activated stroma [6] create difficulties in planning and targeting treatment. An inefficient immune response within the tumour has also been described. This takes places through impaired signalling pathways, such as KRAS, which regulate the growth factor and cytokine milieu as well as immune cell recruitment [6,7]. A better understanding of these pathways could identify specific biomarkers, allowing for earlier and more accurate diagnosis as well as possible therapeutic targets.

Matrix metalloproteinases (MMPs) are proteolytic enzymes associated with maintaining tissue allostasis through the modulation and degradation of the extracellular matrix (ECM). The physiological functions of MMPs include developmental processes, morphogenesis, remodelling and tissue repair as well as inflammation and signalling pathways [8,9]. MMPs are dysregulated in various pathological states such as cancer, arthritis, cardiovascular disease and central nervous system damage [8]. In cancer, MMPs have been described as regulating the epithelial-to-mesenchymal transformation, affecting invasion and metastasis through ECM and basement membrane modulation, altering the growth factor and cytokine environment by revealing molecules embedded in the ECM and cell membranes and through the regulation of apoptosis [10,11].

MMP-8 or collagenase-2 is a collagenase expressed mainly by neutrophils but also by endothelial cells [12] and macrophages [13]. MMP-8 takes part in tissue remodelling both in inflammation and cancer since it can act as a modulator of the immune response as well. In human cancer, results differ since both tumour-promoting and -suppressing qualities occur depending on the tissue of origin [14]. Elevated tissue MMP-8 levels have been identified in squamous cell carcinomas of the head and neck with no prognostic value [15] and ovarian cancer where expression predicted poor prognosis [16]. As such, serum levels may predict prognosis in colorectal [17,18] and gastric cancers [19]. The prognostic value of MMP-8 in PDAC, however, has not been studied extensively.

Polymorphonuclear cells (PMNs) are involved in the innate immune system and can carry both tumourigenic and antitumour effects. Neutrophils, the largest subgroup of PMNs, have been associated with cytotoxicity against cancer cells and reducing metastasis [20]. Neutrophils can, however, also promote cancer progression by affecting transformation, tumour growth and invasion as well as immunosuppression [21]. MMP-8’s contribution to neutrophil recruitment is complex since it is involved in the attraction of cells during the acute phase, but also in resolving inflammation and preventing it from becoming chronic [22]. MMP-8 deficiency in mice has been reported to delay neutrophil recruitment to inflammation sites, which might contribute to sustained and inefficient inflammation [23]. The connection between chronic inflammation and tumourigenesis through cytokines, growth factors and genomic instability inducing factors (reactive oxygen species) is evident [24].

C-reactive protein (CRP) is an acute-phase protein and circulating marker of inflammation. Systemic inflammation appears to correlate with a worse prognosis in various cancers [25]. In PDAC, for instance, an elevated CRP level has been associated with an unfavourable prognosis [26]. MMPs are known mediators of inflammatory processes at the tumour site which may eventually lead to systemic inflammation and CRP elevation.

In this study, we aimed to examine the expression of MMP-8 in PDAC tissue and the amount of MMP-8-positive polymorphonuclear cells (PMNs) in the tumour area. We evaluated the relationships between clinicopathological data and outcomes as well as associations with systemic inflammation marked by an elevated plasma CRP level.

## 2. Results

### 2.1. Immunohistochemical Staining of MMP-8 in Cancer Cells, Number of Tumour-Associated MMP-8-Positive PMNs and Plasma CRP Levels

**Figure 1:**
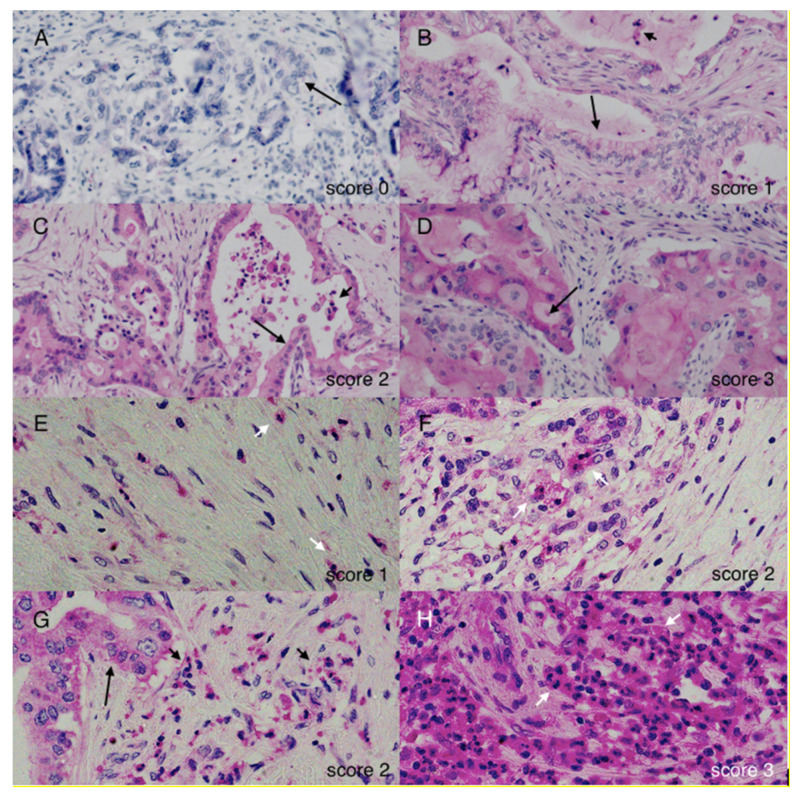
Staining patterns. Negative matrix metalloproteinase 8 (MMP-8) immunostaining in (**A**) scored as 0. Positive MMP-8 immunostaining in (**B**–**D**) scored as 1–3, respectively. MMP-8-positive polymorphonuclear cells (PMNs) in the tumour area scored as 1 in (**E**), 2 in (**F**) and (**G**) and 3 in (**H**). Long arrows indicate the location of cancer cells, while short arrows indicate PMNs.

A total of 141 patients with positive MMP-8 immunohistochemistry scores and sufficient information on clinicopathological data were included in the statistical analysis. Immunopositivity for MMP-8 occurred in the tumour cell cytoplasm with mild nonspecific background staining of the extracellular space in some of the samples. Staining in the cancer cells was clearly distinguishable, and MMP-8 expression was strong in PMNs (Figure 1).

A strong MMP-8 staining intensity was observed in 19 patients (13%), a moderate intensity in 58 patients (41%), a weak intensity in 52 patients (37%) and stained negatively in 12 patients (8.5%). The number of MMP-8-positive PMNs was high in 5 patients (3.5%), moderate in 22 patients (16%), low in 55 patients (39%) and absent in 59 patients (42%). Among those patients with available data on plasma CRP levels, 97 (78%) had a low CRP level (<10 mg/L) and 27 (22%) an elevated CRP level (≥10 mg/L).

### 2.2. Association of MMP-8 Expression, Tumour-Associated MMP-8-Positive PMNs and Plasma CRP Level with Clinicopathological Characteristics

**Figure 2:**
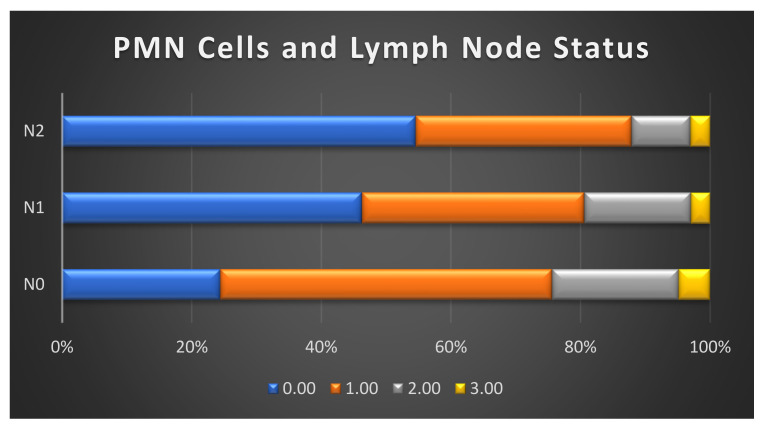
Association between tumour-associated MMP-8 positive polymorphonuclear cells (PMNs) and lymph node status. 0 = no PMNs; 1 = single PMNs; 2 = moderate number of PMNs; 3 = large number of PMNs.

We found no significant correlation between the MMP-8 expression and clinicopathological characteristics. The absence of tumour-associated MMP-8-positive PMNs was associated with the presence of lymph node metastasis (*p* = 0.003; Table 1; Figure 2) as well as perivascular invasion (*p* = 0.010). In addition, the presence of PMNs was more common in female patients (*p* = 0.001; Table 1). We also observed a weak negative correlation between MMP-8 expression and CRP (*p* = 0.011; Table 1). There was no statistically significant correlation between CRP and MMP-8 positive PMNs (Table S1).

### 2.3. Association of MMP-8 Expression, Tumour-Associated MMP-8-Positive PMNs and the Plasma CRP Level with Survival

**Figure 3:**
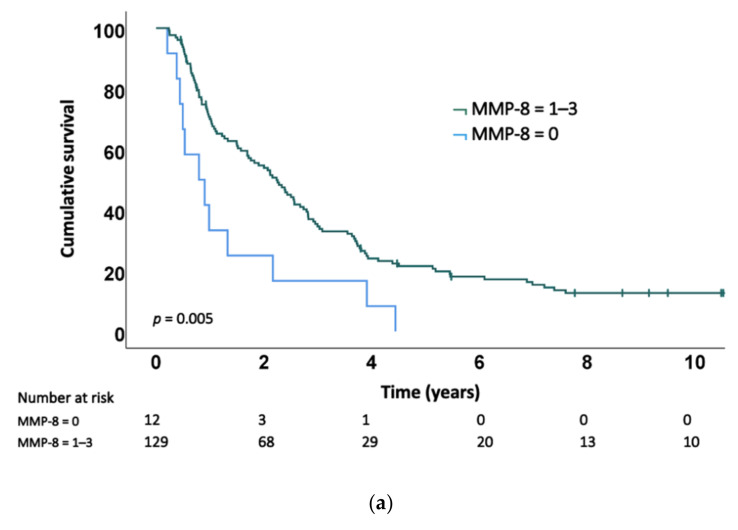

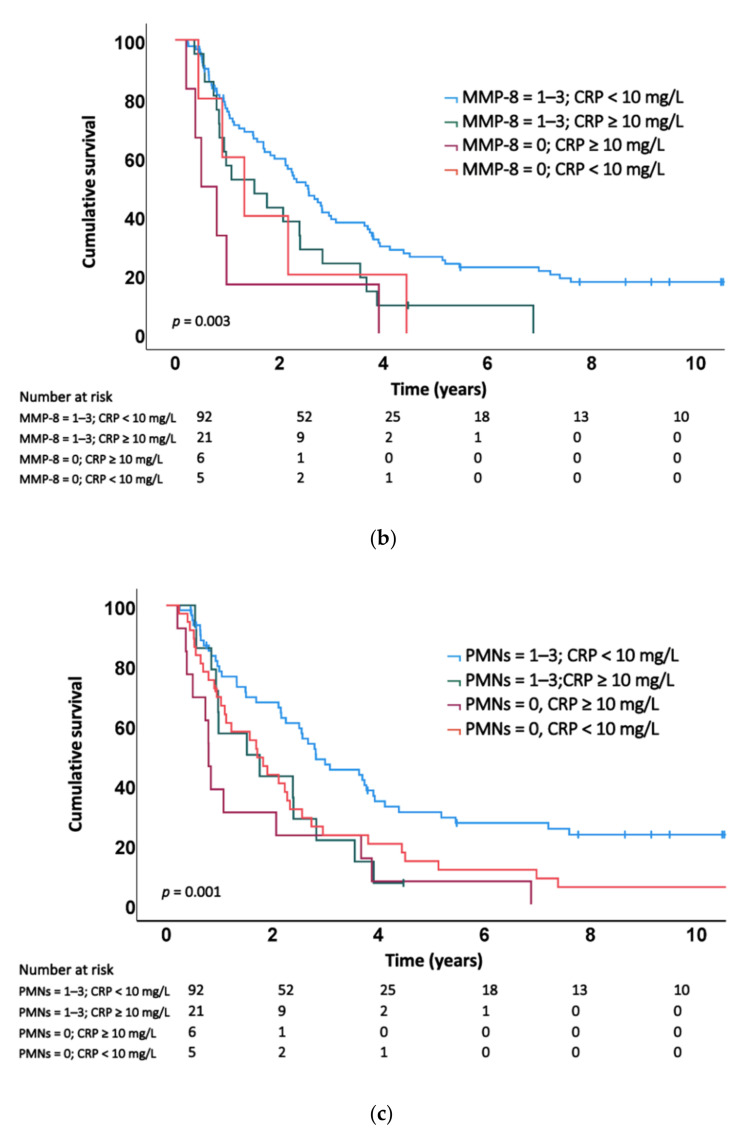
Kaplan–Meier curves. (**a**) Survival in all patients, grouped by matrix metalloproteinase 8 (MMP-8) expression in cancer cells (*p* = 0.005); (**b**) survival in all patients, grouped by MMP-8 expression in cancer cells and plasma C-reactive protein (CRP) level (*p* = 0.003); (**c**) survival in all patients, grouped by MMP-8 positive polymorphonuclear cell (PMN) presence in the tumour and plasma CRP level (*p* = 0.001).

In the Kaplan–Meier analysis, a positive MMP-8 staining score (1–3) in cancer cells predicted a prolonged survival compared to a negative staining score (Figure 3a). We also analysed the combined prognostic value of MMP-8 and plasma CRP levels. Patients with a positive tumoural MMP-8 stain and a low CRP level exhibited the best prognosis, whereas those with a negative MMP-8 stain and elevated CRP level exhibited the worst prognosis (Figure 3b). The presence of tumour-associated MMP-8-positive PMNs combined with a low CRP level also predicted a better survival (Figure 3c).

In the univariable analysis, patients with a weak to strong (1–3) MMP-8 cancer cell expression score exhibited a better survival (hazard ratio (HR) = 0.43; 95% confidence interval (CI) 0.24–0.79; *p* = 0.006). A negative MMP-8 score and an elevated CRP level predicted a worse prognosis (HR = 3.45; 95% CI 1.49–8.01; *p* = 0.004) compared to a positive MMP-8 score and a low CRP level. The absence of tumour-associated MMP-8-positive PMNs together with an elevated CRP level associated with an unfavourable prognosis (HR = 2.99; 95% CI 1.60–5.59; *p* = 0.001) compared to a positive PMN score and a low CRP level (Table 2). Furthermore, the presence of MMP-8-positive PMNs served as a positive prognostic factor in the univariable analysis (HR = 0.53; 95% CI 0.37–0.76; *p* = 0.001). Patients with an elevated CRP level exhibited a worse prognosis compared to those with a low CRP level (HR = 1.93; 95% CI 1.23–0.03; *p* = 0.005).

The multivariable analysis confirmed that the MMP-8 expression level in cancer cells represents an independent positive prognostic factor (HR = 0.33; 95% CI 0.16–0.68; *p* = 0.003; Table 3). CRP was an independent negative prognostic factor (HR = 2.25; 95% CI 1.34–3.76; *p* = 0.002; Table 3) as previously described by Salmiheimo et al., using the same patient cohort [26]. In the multivariable analysis, we detected no statistically significant survival advantage when considering tumour-associated MMP-8-positive PMNs as an independent prognostic factor. We also considered the combination of MMP-8 and the CRP level as a prognostic factor in the multivariable analysis adjusted by age, sex, T and N status, perivascular invasion, adjuvant therapy and the carbohydrate antigen 19-9 (CA19-9) level. A negative MMP-8 score combined with an elevated CRP level predicted a poor prognosis compared to a positive staining score and a low CRP level (HR = 6.95; 95% CI 2.69–17.93; *p* < 0.001). Furthermore, the absence of tumour-associated MMP-8-positive PMNs together with an elevated CRP level predicted a worse prognosis compared to a positive PMN score and a low CRP level (HR = 3.17; 95% CI 1.60–6.30; *p* = 0.001; adjusted for age, sex, T and N status, perivascular invasion, adjuvant therapy and CA19-9).

## 3. Discussion

We found that MMP-8 immunoexpression in cancer cells combined with a low plasma CRP level (<10 mg/L) is a strong predictor of a favourable outcome in PDAC, whereas no MMP-8 expression and an elevated CRP level (≥10 mg/L) predicts a poor prognosis. Moreover, MMP-8 expression in cancer cells served as an independent positive prognostic factor in PDAC. Tumour-associated MMP-8-positive PMNs combined with a low CRP level associated with a better prognosis compared to the absence of PMNs and an elevated CRP level. In addition, the absence of tumour-associated MMP-8-positive PMNs correlated with lymph node metastasis.

To our knowledge, no previous studies have evaluated the combined prognostic value of CRP levels and MMP-8 expression in PDAC. In colorectal cancer (CRC), a link between serum MMP-8 and CRP levels has been reported [17,18]. Interestingly, serum MMP-8 levels served as a negative prognostic marker among CRC patients with no systemic inflammatory response [18]. MMP-8 reduces the invasive capacity of cancer and enhances the immune response by regulating neutrophil recruitment, as illustrated in animal and in vitro studies [27,28]. Chemotaxis alterations induced by MMP-8 also affect T-cell responses dysregulated in PDAC [7,20,29]. These immunoregulatory mechanisms also provide a possible explanation for the positive prognostic value of the combination of MMP-8 expression and low CRP levels.

According to our findings, a high number of MMP-8-positive PMNs in a tumour together with low CRP levels indicate a better prognosis compared to the absence of PMNs and an elevated CRP level. Interestingly, a high neutrophil count typically associates with poor survival in cancer, although findings appear contradictory [30]. In PDAC, a high neutrophil-to-lymphocyte ratio (NLR) in peripheral blood predicts a poor prognosis [31]. However, circulating neutrophils indicate a systemic inflammation not efficiently targeting the tumour. PDAC is associated with an immunosuppressive microenvironment with inflammatory cells and cytokines promoting cancer progression [32]. In our study, the presence of PMNs may indicate an active, local immune response and, thus, a potentially better prognosis. Tumour-infiltrating PMNs have been associated with cytotoxicity against tumour cells and a better prognosis in women with gastric cancer [33]. In CRC, a local immune response and tumour-infiltrating FOXP3+ T-regulatory cells inversely correlated with a systemic inflammatory response [34], which associated with a poor prognosis in various cancers [25]. This supports our theory that the quality of the immune response and the inflammatory cell type significantly affect cancer progression and that an effective, local immune response serves as a protective factor. However, the results differ depending on the origin and histology of the cancer [35], and apparently tumour-associated neutrophils (TANs) exhibit both antitumour and tumourigenic properties. Our results suggest that antitumour mechanisms may be more important in PDAC. Despite the contribution of MMP-8 in regulating neutrophil migration, we found no significant correlation between tumoural MMP-8 and the PMN count. This might result from complex interactions in the tumour microenvironment not yet fully understood. Overall, no comprehensive studies exist with a setting similar to ours regarding tumour-associated PMNs in PDAC and, thus, their prognostic value requires further research.

Our results suggest that the MMP-8 expression serves as an independent predictor of a better prognosis in PDAC. MMP-8 expression in pancreatic cancer has not been studied extensively. In a small study on PDAC biomarkers among 19 patients, the overexpression of MMP-8 and -9 was associated with a shorter survival (<12 months) [36]. These findings differ from ours and may result from a different technique (proteomic analysis) and the exceedingly low number of cases. Another study among 45 PDAC patients failed to show any correlation between MMP-8 levels and patient survival, although MMP-8 staining intensity in the tumour was high compared to normal pancreatic tissues [37]. The differences in these results might be explained by the relatively small sample sizes considering neither one of these previous studies focused specifically on MMP-8. In other cancers of the gastrointestinal tract, the prognostic role of MMP-8 remains controversial. A high MMP-8 serum level predicts a worse prognosis in hepatocellular carcinoma [38]. In addition, elevated serum levels associated with the tissue inhibitors of matrix metalloproteinases (TIMPs) appear to predict a poor prognosis in CRC [18] and gastric cancer [19]. The connection between serum MMP-8 levels and systemic inflammation has also been reported in CRC patients [17]. These findings are difficult to compare with ours since MMP-8 in tissue and circulation appear to exert differing roles. Cytokines and enzymes may also have varying biological functions throughout different stages of disease [32]. Nevertheless, elevated serum levels appear to associate with pathological processes more often while tumour expression may enhance the local immune response serving as a protective factor in some cancers.

According to our study, tumour-associated MMP-8-positive PMNs are an independent positive prognostic factor in the univariable analysis, but the multivariable analysis did not confirm this result. In a small study among 23 PDAC patients, TANs served as a negative prognostic factor [39]. The difference in these results might be explained by a small sample size compared to ours as well as the use of a different molecular marker (CD177) for neutrophils, since we did not study the total TAN but only MMP-8-positive TANs. Additionally, interactions between different subsets of immune cells may affect their individual prognostic values in cancer [40], complicating the evaluation of causal connections between tumour-associated PMNs and prognosis. We also found that there is an inverse correlation between the PMN count at the tumour site and lymph node metastasis. Patients with lymph node metastasis (N2) were less likely to have MMP-8-positive PMNs in the tumour and vice versa. This indicates that the TAN may not matter, while the MMP-8 activity does, suggesting that MMP-8 may have a favourable effect on survival and act locally as a tumour suppressor. Furthermore, an elevated NLR preoperatively has been associated with lymph node metastasis in PDAC [41], but NLR is not comparable to tumour-infiltrating neutrophils. In EBV-associated gastric carcinoma, the absence of TANs reportedly correlates with lymph node metastasis [42], supporting our hypothesis that PMNs at the tumour site may have qualities that suppress cancer progression.

Although MMP-8 immunoexpression in the tumour seems to represent an independent prognostic factor, the combination of the CRP level and MMP-8 expression offers more precise prognostic information. The well-established connection between inflammation and cancer seems to be bidirectional with systemic inflammation altering the tumour microenvironment and the tumour modulating the immune response through cytokine and chemokine production [43]. Our results not only demonstrate the role of tissue MMP-8 expression in cancer cells as a positive prognostic factor in PDAC, but also offer a possible protective mechanism through the prevention of chronic inflammation.

The strengths of this study lie in the large cohort size with a long follow-up time and good coverage of clinicopathological data and endpoints. The weaknesses include using a tissue microarray (TMA), since only a small proportion of the cancer is analysed. However, the TMA technique enables representative sample evaluation from large patient cohorts efficiently and four cores per patient minimised the risk of lost information due to sampling. Moreover, some clinicopathological characteristics were unavailable leading to the exclusion of some patients and variables. While the evaluation of immunohistochemical (IHC) staining intensity scores is subjective, the distinction between negative and positive staining results was unambiguous. The opportunity to use a patient cohort with no neoadjuvant treatment permitted us to observe the tumour biology prior to treatment-induced alterations.

## 4. Materials and Methods

### 4.1. Patients

The study population consists of PDAC patients surgically treated between 2002 and 2011 in the Department of Surgery, Helsinki University Hospital, Finland. The cohort analysed included 147 PDAC patients with stage I–III disease undergoing upfront surgery who received no neoadjuvant treatment. Six patients were excluded due to a lack of representative cancer tissue in the TMA, resulting in 141 patients for analysis. Information on plasma CRP levels was available for 124 patients. Survival data and causes of death information were obtained from hospital records, the Finnish Population Registry and Statistics Finland.

### 4.2. Preparation of Tumour Tissue Microarrays, Immunohistochemistry and the Measurement of the Plasma C-Reactive Protein Level

Formalin-fixed and paraffin-embedded (FFPE) samples from resected tumour tissue were collected from the archives of the Department of Pathology, Helsinki University Hospital. Samples were stained with haematoxylin and eosin and analysed by experienced pathologists to confirm the histopathological diagnosis of PDAC.

Multipunch tissue microarray (TMA) blocks were prepared from the PDAC tissue sample paraffin blocks. Six cores 1.0 mm in size were cut from different tumour areas from each sample and replaced on a new paraffin block. For the immunohistochemistry, TMA blocks were cut into 4 μm sections and mounted on a positively charged glass (TOMO). Saukkonen et al., previously described the preparation of the samples and the TMA technique in more detail [44].

For MMP-8 staining and evaluation of PMNs, samples were deparaffinised with xylene and rehydrated in solutions with gradually decreasing ethanol concentrations using Sakura Tissue-Tek DRS (Sakura Finetek USA, Inc., Torrance, CA, USA). Antigen retrieval was performed using Agilent Dako’s Pre-Treatment module (pH 9) (Agilent, Santa Clara, CA, USA). An EnVision Flex peroxidase blocking agent (Agilent, Santa Clara, CA, USA) was used to prevent the nonspecific binding of antibodies.

Samples were incubated in the primary MMP-8 antibody solution (dilution 1:400, Dako REAL antibody diluent S2022, described by Hanemaaijer et al., 1997 [12] and Prikk et al. [45]) overnight in +4 °C. The specificity of the MMP-8 antibody was tested by Hanemaaijer et al., through the addition of excessive amounts of different MMPs during Western blotting with the MMP-8 antibody, which recognised no other MMPs than MMP-8 [12].

EnVision Flex/HRP SM802 (Agilent, Santa Clara, CA, USA) was used as a secondary antibody with an incubation time of 30 min. DAB and magenta were used as chromogens and haematoxylin was used to counterstain the samples. A tonsil hyperplasia sample served as the positive control. We used an Autostainer 480S (Lab Vision Corp., Fremont, CA, USA) for the staining process as described previously by Saukkonen et al. [44].

The plasma CRP levels of patients were determined previously with an immunofluorometric assay using a monoclonal CRP antibody as described by Salmiheimo et al. [26].

### 4.3. Evaluation of Stainings

The samples were evaluated using two parameters, namely, the intensity of the cytoplasmic MMP-8 staining in the cancer cells and the number of MMP-8-positive PMNs in the tumour area. PMN cells were identified by their MMP-8 expression and morphology. The cytoplasmic staining intensity was scored as negative (0), weakly positive (1), moderately positive (2) and strongly positive (3). A scale of 0–3 was used for the PMN cell number as well. The median of the scores from each of the four TMA spots was used in the analysis as the most representative value. Samples were scored by two independent investigators (M.K. and J.H.) and the evaluation was performed without knowledge of any clinical data or patient survival. Differences in scoring were resolved through discussion to establish a consensus score.

### 4.4. Statistical Analysis

Data are presented as number of cases and proportion of cases or as median and interquartile range (IQR). The Shapiro Wilk’s test was used to determine if continuous variables followed a normal distribution. Survival time was calculated from the time of surgery until death or to the end of follow-up (6 January 2020). A disease-specific event was defined as death from PDAC. The Kaplan–Meier analysis was used to assess survival and to obtain survival curves, and the log-rank test was used to compare survival between different groups. The Cox proportional hazards model was used for uni- and multivariable survival analyses. The assumption of a constant hazard ratio (HR) over time was assessed using scaled Schoenfeld residuals. Interaction terms were considered, but we identified no significant interactions in the multivariable models after Bonferroni correction for multiple comparisons. The Spearman’s correlation coefficient was calculated to assess bivariate correlations. Two-tailed tests were used and we considered *p* < 0.05 as statistically significant. All statistical analyses were performed with SPSS (v.25, SPSS, Inc., an IBM Company, Chicago, IL, USA) or with R (v.4.0.0, Foundation for Statistical Computing, Vienna, Austria).

## Figures and Tables

**Table 1:** Association of cancer cell matrix metalloproteinase 8 (MMP-8) expression in cancer cells, the number of tumour-associated MMP-8 positive polymorphonuclear cells (PMNs) and the plasma C-reactive protein (CRP) level with clinicopathological characteristics.

	Cancer Cell MMP-8		*p* Value	PMNs		*p* Value	CRP Levels		*p* Value
	Negative (0)	Positive (1–3)		Negative (0)	Positive (1–3)		Low (<10 mg/L)	High (≥10 mg/L)	
*n* (%)	12 (8.5)	129 (86.5)		59 (41.8)	82 (58.2)		97 (78.2)	27 (21.8)	
Age, in years			0.208			0.135			0.107
<65	5 (7.2)	64 (92.8)		29 (42.0)	40 (58.0)		50 (82.0)	11 (18.0)	
≥65	7 (9.7)	65 (90.3)		30 (41.7)	42 (58.3)		47 (74.6)	16 (25.4)	
Sex			0.168			0.001			0.170
Male	8 (10.4)	69 (89.6)		41 (53.2)	36 (46.8)		55 (77.5)	16 (22.5)	
Female	4 (6.3)	60 (93.8)		18 (28.1)	46 (71.9)		42 (79.2)	11 (20.8)	
T1	1 (7.1)	13 (92.9)	0.171	2 (14.3)	12 (85.7)	0.036	12 (100.0)	0 (0.0)	
T2	7 (7.6)	85 (92.4)		41 (44.6)	51 (55.4)		63 (78.8)	17 (21.2)	
T3	4 (11.4)	31 (88.6)		16 (45.7)	19 (54.3)		22 (68.8)	10 (31.2)	
N0	4 (9.8)	37 (90.2)	0.163	10 (24.4)	31 (75.6)	0.003	28 (84.8)	5 (15.2)	
N1	5 (7.5)	62 (92.5)		31 (46.3)	36 (53.7)		43 (72.9)	16 (27.1)	
N2	3 (9.1)	30 (90.9)		18 (54.5)	15 (45.5)		26 (81.3)	6 (18.7)	
Grade			0.094			0.113			
1	0 (0.0)	15 (100.0)		6 (40.0)	9 (60.0)		9 (75.0)	3 (25.0)	
2	8 (8.9)	82 (91.9)		37 (41.1)	53 (58.9)		61 (76.3)	19 (23.7)	
3	3 (13.0)	20 (87.0)		11 (47.8)	12 (52.2)		17 (81.0)	4 (19.0)	
Perineural invasion			0.249			0.088			
No	4 (9.1)	40 (90.9)		16 (36.4)	28 (63.6)				
Yes	8 (8.7)	84 (91.3)		42 (45.7)	50 (54.3)				
Perivascular invasion			0.266			0.010			
No	9 (9.0)	91 (91.0)		37 (37.0)	63 (63.0)				
Yes	3 (8.1)	34 (91.9)		22 (59.5)	15 (40.5)				
ASA score			0.190			0.102			
1	0 (0.0)	3 (100.0)		0 (0.0)	3 (100.0)		2 (66.7)	1 (33.3)	
2	5 (12.2)	36 (87.8)		18 (43.9)	23 (56.1)		33 (84.6)	6 (15.4)	
3	5 (9.8)	46 (90.2)		23 (45.1)	28 (54.9)		31 (68.9)	14 (31.1)	
4	1 (20.0)	4 (80.0)		2 (40.0)	3 (60.0)		3 (75.0)	1 (25.0)	
CRP			0.011			0.102			
<10 mg/L	5 (5.2)	92 (94.8)		36 (37.1)	61 (62.9)				
≥10 mg/L	6 (22.2)	21 (77.8)		13 (48.1)	14 (51.9)				

**Table 2:** Univariable analysis of relative risk of death from pancreatic ductal adenocarcinoma (PDAC) by matrix metalloproteinase 8 (MMP-8) expression level in cancer cells, the amount of tumour-associated MMP-8 positive polymorphonuclear cells (PMNs), the plasma C-reactive protein (CRP) level and clinicopathological characteristics.

	Hazard Ratio	95% CI	*p* Value
Age	1.16	0.81–1.66	0.420
Sex	0.87	0.60–1.24	0.431
T1	1 (reference)		
T2	1.16	0.63–2.13	0.641
T3	1.52	0.78–2.97	0.223
N0	1 (reference)		
N1	1.47	0.95–2.28	0.084
N2	2.62	1.59–4.33	<0.001
Grade			
1	1 (reference)		0.486
2	0.90	0.52–1.57	0.707
3	1.21	0.62–2.38	0.573
Perivascular invasion	1.71	1.14–2.55	0.009
Perineural invasion	1.06	0.72–1.55	0.773
Adjuvant therapy	0.88	0.62–1.26	0.485
Tumour size	1.32	0.92–1.89	0.132
CA19-9 (kU/l, log_10_)	1.28	1.08–1.53	0.006
CRP ≥ 10 vs. <10 mg/L	1.93	1.23–3.03	0.005
MMP-8 = 1–3 vs. 0	0.43	0.24–0.79	0.006
PMNs = 1–3 vs. 0	0.53	0.37–0.76	0.001
MMP-8 = 1–3, CRP < 10 mg/L	1 (reference)		0.005
MMP-8 = 0, CRP < 10 mg/L	1.98	0.80–4.94	0.141
MMP-8 = 1–3, CRP ≥ 10 mg/L	1.78	1.07–2.94	0.025
MMP-8 = 0, CRP ≥ 10 mg/L	3.45	1.49–8.01	0.004
PMNs = 1–3, CRP < 10 mg/L	1 (reference)		0.001
PMNs = 0, CRP < 10 mg/L	1.86	1.19–2.93	0.007
PMNs = 1,2,3, CRP ≥ 10 mg/L	2.06	1.10–3.87	0.025
PMNs = 0, CRP ≥ 10 mg/L	2.99	1.60–5.59	0.001

**Table 3:** Multivariable analysis of the relative risk of death from pancreatic ductal adenocarcinoma (PDAC) by matrix metalloproteinase 8 (MMP-8) expression level in cancer cells, tumour-associated MMP-8 positive polymorphonuclear cells (PMNs) and plasma C-reactive protein (CRP) and clinicopathological characteristics.

	Hazard Ratio	95% CI	*p* Value
Age	1.43	0.93–2.20	0.101
Sex	1.05	0.67–1.66	0.829
T1	1 (reference)		0.317
T2	0.61	0.29–1.30	0.203
T3	0.52	0.22–1.22	0.132
N0	1 (reference)		0.001
N1	1.96	1.09–3.53	0.025
N2	4.00	1.98–8.10	<0.001
Perivascular invasion	1.63	1.02–2.61	0.043
Adjuvant therapy	0.62	0.40–0.97	0.038
CA19-9 (kU/l, log_10_)	1.26	1.00–1.58	0.047
CRP ≥ 10 vs. <10 mg/L	2.25	1.34–3.76	0.002
MMP-8 = 1–3 vs. 0	0.33	0.16–0.68	0.003
PMNS = 1–3 vs. 0	0.75	0.48–1.16	0.196

## Data Availability

Data availability requests can be made to the corresponding authors.

## Supplementary Materials

The following are available online at https://www.mdpi.com/article/10.3390/ijms23063314/s1.
