# Supplementary material for: Matrix Metalloproteinase 8 Expression in a Tumour Predicts a Favourable Prognosis in Pancreatic Ductal Adenocarcinoma
Source: Int J Mol Sci. 2022 Mar 18;23(6):3314. doi: 10.3390/ijms23063314 (PMC8951094; doi:10.3390/ijms23063314)
Supplement: Supplementary file 1 [file ijms-23-03314-s001.zip › ijms-1555115-supplementary.pdf]

Table S1: Non-parametric correlations between C-reactive protein (CRP), matrix metalloproteinase 8 (MMP-8) expression in cancer cells and tumour-associated MMP-8 positive polymorphonuclear cells (PMNs).

	**CRP**	***p*-value**
CRP (mg/L, continuous)	1	
MMP-8 (0,1,2,3 scores)	0.13 (95% CI -0.05-0.31)	0.149
PMNs (0,1,2,3 scores)	-0.08 (95% CI -0.26-0.10)	0.378
